# Medial Rectus Tendon Elongation with Bovine Pericard (Tutopatch®) in Thyroid-Associated Orbitopathy: A Long-Term Follow-Up including Oculodynamic MRI

**DOI:** 10.1155/2018/1294761

**Published:** 2018-07-24

**Authors:** Monika Wipf, Britt-Isabelle Berg, Anja Palmowski-Wolfe

**Affiliations:** ^1^University Eye Hospital, University of Basel, Mittlere Strasse 91, 4031 Basel, Switzerland; ^2^Department of Cranio-Maxillofacial Surgery, University of Basel, Spitalstrasse 21, 4031 Basel, Switzerland; ^3^Division of Oral and Maxillofacial Radiology, Columbia University Medical Center, 622 W. 168th St., New York, NY 10032, USA

## Abstract

**Introduction:**

To assess long-term efficacy of bimedial rectus tendon elongation with Tutopatch in thyroid-associated orbitopathy (TAO).

**Materials and Methods:**

Retrospective chart review of 5 patients with TAO undergoing bimedial rectus recession with Tutopatch tendon elongation between 2009 and 2015. We analyzed horizontal squint angles, motility, field of binocular single vision, dose effect of surgery, and when possible oculodynamic MRI (OD-MRI). Dose effect and motility were compared to 4 TAO patients with conventional bimedial recession.

**Results and Discussion:**

In the Tutopatch group, preoperative angles ranged from 14 to 120∆ (prism diopters) at distance and 12–120∆ at near. Mean dose effect was 3.63∆/mm for the distance and 3.43∆/mm for the near angle. All patients were orthotropic at final FU (ranging from 1 to 10 years). OD-MRI showed the elasticity of Tutopatch. In the conventional recession group, preoperative angles ranged between 18 and 35∆ at distance and 12–33∆ at near. At final FU, 2 patients had reverted to their underlying microesotropia <2∆, 1 patient was orthophor, and one was reoperated for a remaining esotropia of 14∆. Dose effect was 2.95∆/mm for the distance and 2.18∆/mm for the near angle. Motility improved in both groups even after 3 months.

**Conclusions:**

Dose effect for medial rectus recessions with Tutopatch in TAO was higher than previously reported, presenting a good alternative to treat large squint angles while preserving good motility.

## 1. Introduction

In patients with thyroid-associated orbitopathy (TAO), inflammation and fibrotic changes of the extraocular muscles may cause restriction of eye movements with strabismus and diplopia. The inferior rectus muscle is most commonly affected, followed by the medial rectus muscle [1, 2]. Restrictive strabismus is corrected by recession of the fibrotic muscle. Generally, the medial rectus muscle may be recessed up to 5 mm and the inferior rectus muscle up to 6 mm without causing additional weakening in the direction of muscle action [3]. In TAO, the mean dose effect (DE) reported for bilateral medial rectus recession is 1.56–1.59°/mm (≈2.7∆/mm) [1]. Thus, in angles exceeding 20° (≈40∆), conventional bilateral recession alone does not yield enough reduction of angle.

Large angles occur more commonly in patients following orbital decompression surgery, as the orbital content may shift to the side following removal of an orbital wall. This is especially common after medial wall recessions [1]. Following orbital decompression, the reported mean DE of a bilateral medial rectus recession is reduced to 1.2°/mm (≈2.1∆/mm), which means that the maximal effect of a bilateral 6.5 mm recession is only 15.6° (≈27.3Δ) [1]. For patients with larger angles, a tendon elongation procedure using bovine pericard, Tutopatch, has been suggested for the inferior rectus muscle [4].

**Figure 1:**
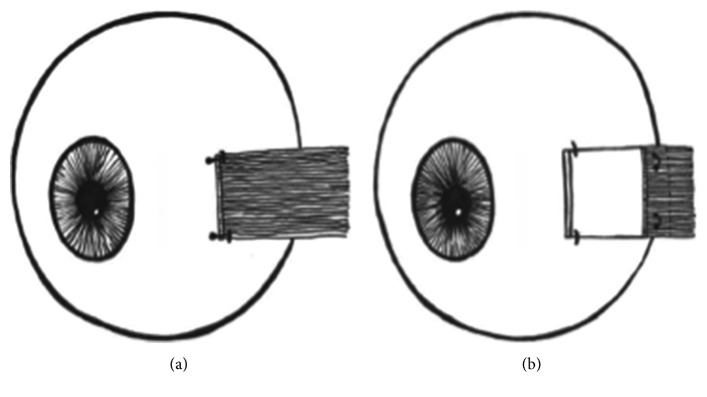
Schematic representation of conventional rectus muscle recession (a) and medial rectus muscle recession with Tutopatch tendon elongation (b). Note that the extraocular muscle comes to lie over Tutopatch, so that there is no direct contact between the tendon stump and the sclera.

This study focuses on the less frequently performed medial rectus recession with tendon elongation using Tutopatch. The technique is discussed elsewhere [4] and schematically represented in Figure 1. Further, we compare the DE of these individuals with those of other patients with TAO that underwent conventional bimedial recession at our hospital. These DEs are compared to those found in the literature. Additionally, we made use of oculodynamic MRI (OD-MRI) performed on four patients of the Tutopatch group to analyze the postoperative motility.

## 2. Materials and Methods

Approval of the Institutional Review Board to undertake this study was obtained in August 2015. The study was conducted in adherence to the Declaration of Helsinki.

The charts of all patients with TAO who underwent bilateral recession of the medial rectus muscle and tendon elongation with Tutopatch between 2006 and 2015 in our institution were retrospectively reviewed.

In addition, the charts of all patients with TAO who underwent a conventional bilateral medial rectus muscle recession between 2006 and 2015 were retrospectively reviewed for comparison. This resulted in a total number of 9 patients, 5 of whom received a tendon elongation with Tutopatch and 4 of whom underwent conventional muscle recession.

### 2.1. Patients

Patient details are summarized in Table 1

1. Patient 1, female, aged 60 at strabismus surgery with tendon elongation. She was diagnosed with TAO 3 years prior to surgery and had had no previous orbital decompression. FU was 84 months.
2. Patient 2A, female, aged 53 at strabismus surgery with tendon elongation. She was diagnosed with TAO 2 years prior to surgery, underwent bilateral orbital decompression consisting of an endonasal osteotomy of the medial orbital wall followed by a resection of the lateral orbital wall via coronary access, and developed a consecutive esotropia. She had also undergone radioiodine treatment. She had had a conventional bilateral recession of the medial rectus muscle 7.5 months prior to tendon elongation with Tutopatch with insufficient effect and a remaining manifest angle of 14∆ (data shown as patient 2B below). In addition to the tendon elongation, the scleral attachment of the right medial rectus muscle was readvanced by 1.5 mm, resulting in a total recession of 6 mm. FU was 130 months.
3. Patient 3, male, aged 43 at strabismus surgery with tendon elongation, was diagnosed with TAO 1 year before surgery. He underwent orbital decompression (same procedure as patient 2, with endonasal medial decompression and lateral orbitotomy via coronary access) with an increase of his squint angle from +20∆ to +120∆. He had an early strabismus surgery one month after systemic treatment with i.v. steroids according to Kahaly et al. [5]. FU was 41 months.
4. Patient 4, male, aged 42 at surgery, was diagnosed with TAO 2 years before strabismus surgery with tendon elongation. He had orbital decompression (same procedure as patients 2 and 3) 1.5 years prior to surgery and also underwent radioiodine therapy. FU was 36 months.
5. Patient 5, female, aged 66 at strabismus surgery with tendon elongation, diagnosed with TAO 1 year prior to surgery underwent orbital decompression of the medial orbital wall only (via endonasal access). This was the only patient in the Tutopatch group with no history of smoking. This case has been described with a 7-month FU in a previous case report [6]. Here, we are able to follow up to 13 months and present her OD-MRI.
6. Patient 6, female, aged 56 at surgery, diagnosed with TAO 1 year prior to conventional strabismus surgery. She had undergone bilateral orbital decompression 15 months previously via endonasal access with ethmoidectomy and underwent bilateral radiation of the orbit. FU was 47 months.
7. Patient 7, female, aged 35 at conventional strabismus surgery. She had no previous radiation of the orbit and was the only patient of the conventional recession group with no history of smoking. She was diagnosed with TAO 1 year before surgery at the age of 34 years, whereas she had been diagnosed with Morbus Basedow at the age of 27 years. She had undergone orbital decompression 1 year before eye muscle surgery. FU was 81 months.
8. Patient 8, female, aged 50 at conventional strabism surgery. She was diagnosed with TAO 3 years prior to surgery and an orbital decompression had been performed 12 months prior to eye muscle surgery, as well as radioiodine therapy two years prior to surgery. FU was 87 months.

Findings obtained preoperatively and at postoperative weeks one and twelve and at final follow-up (FU) were evaluated. Orthoptic measures included were Snellen visual acuity, squint angle at near and distance, adduction and abduction measured with the Kestenbaum limbus test [7], binocular single vision (Harms tangent screen), and stereo vision (Bagolini, Lang, TNO, and Titmus test) [3].

We made use of OD-MRI performed on four patients of the Tutopatch group to further analyze postoperative motility. Details of the OD-MRI technique have been described by Berg et al. [8].

TAO-specific data analyzed were as follows: presence of diplopia and clinical activity score (CAS) [9], information on previous surgeries (extraocular muscles and orbital decompression), previous and current treatment of the TAO (steroids)/Graves' disease (GD) (thyroid surgery and radioiodine treatment), patient's age at onset of TAO, and history of tobacco use.

Collected surgical data included the amount of muscle recessed, length of tendon elongation, complications reported, and patient satisfaction.

The dose effect was calculated separately for the angles at distance and near fixation. DE = (presurgical manifest angle in prism diopters − angle at last follow-up in prism diopters)/total recession in millimeters.

## 3. Results

### 3.1. Patients with Bimedial Recession with Tutopatch

All patients had undergone intravenous steroid treatment according to Kahaly et al. [5] at some point of their TAO.

Prior to strabismus surgery with Tutopatch, patients did not show signs of clinical activity for at least six months, with the exception of P3 and P5 with large incapacitating squint angles.

**Figure 2:**
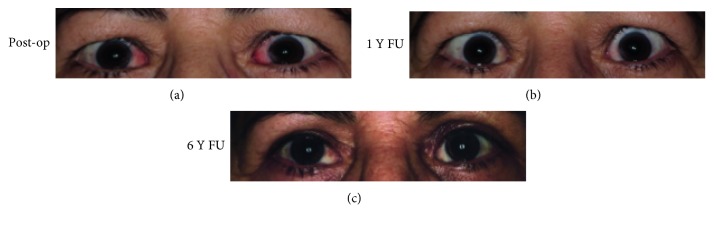
Patient 2 after surgery with Tutopatch (a) and 1 year later (b). (c) The same patient following lid surgery to correct her retraction of the upper eye lid.

**Figure 3:**
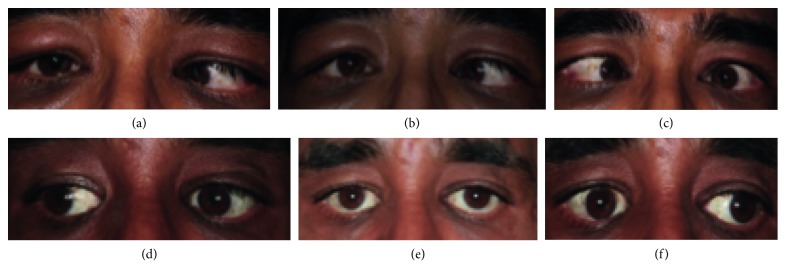
Images showing patient 3 before surgery in rightgaze (a), primary position (b), and leftgaze (c). The patient 4 years after bimedial rectus recession with Tutopatch in rightgaze (d), in primary position (e), and in leftgaze (f). Considering the large recession of 17.5 mm per medial rectus muscle, the patient shows excellent motility with some restriction of adduction on the right eye > left eye.

The mean age at the time of surgery was 52.8 years. Follow-up ranged from 13 to 130 months. For each patient, Table 2 describes the development of the horizontal deviation at distance, the dosage, and DE of surgery. Figures 2 and 3 show examples of individual patients. Before surgery, all patients showed a manifest esodeviation with angles between 14∆ (prism diopters), in the patient with previous conventional medial recession, and 120∆ at distance. At near fixation, angles ranged between 12 and 120∆. This compared to a mean postoperative manifest deviation of 0∆ at distance as well as at near fixation. The mean length of tendon elongation per muscle was 7.55 mm, ranging from 3.75 mm to 13.5 mm with a mean total recession of 9.7 mm per muscle, ranging from 2.25 mm to 17.5 mm. The mean DE at the last FU was 3.63, ranging from 2.33 to 4.59∆/mm for the distance angle and 3.43 ranging from 2 to 4.52∆/mm for the near angle.

Table 3 shows the development of the horizontal motility: at baseline, mean abduction per eye was 3.05 mm (range: 0–6 mm). At the 3-month FU, mean abduction had increased by about 2 mm. At final FU, abduction had increased further to 5.7 (2.5–8.5) mm.

Mean adduction at baseline was 7.95 mm (range: 5–11 mm). As expected this was decreased at the 3-month FU by about 2.6 mm. At final FU, adduction improved slightly to 5.9 (2–9) mm.

### 3.2. Conventional Bimedial Recession Group

Mean age at surgery was 48.25 years. The patients were followed for a mean of 55.6 (7.5–87) months (Table 4). Mean preoperative esodeviation was 27 (18–35)∆ at distance and 21 (12–33)∆ at near fixation. At the last follow-up, two patients with underlying microesotropia still had a manifest deviation, ranging from 0 to 2∆ (Table 4). In patient 2B, a manifest angle of 14∆ at distance remained, which resulted in further recession of the medial recti with Tutopatch (=patient 2A). Total recession was on average 8 (7–10.5) mm. The mean DE was 2.95∆/mm at distance and 2.18∆/mm at near fixation.

Preoperative mean abduction and mean adduction in patients 6–8 were 6.0 and 9.38 mm, respectively. At three months, abduction had increased to 6.82 mm, and adduction had decreased to 8 mm. At the final follow-up, abduction increased further to 7.56 mm, and adduction increased as well to 8.81 mm (Table 3).

## 4. Discussion

Our results confirm that tendon elongation of the medial rectus muscle with Tutopatch is a valid option in patients with severe restriction of ocular motility due to TAO.

Esser et al. [4] have reported on the recession of the inferior rectus muscle using tendon elongation with Tutopatch in TAO with good results (FU up to 6 months). Eckstein et al. [10] reported on bilateral medial rectus recession with Tutopatch tendon elongation in 30 patients with TAO following different types of orbital decompression. In that study, patients were followed up to 3 months. While we report on fewer patients, we report a longer follow-up of up to 10 years and the inclusion of OD-MRI videos that allow appreciation of extraocular muscle motility in vivo.

**Figure 4:**
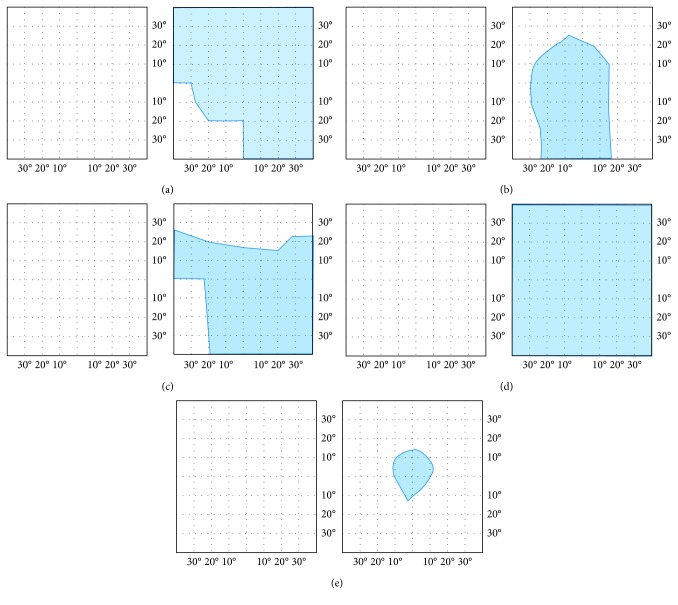
The field of binocular single vision (FBSV, blue area) of the patients with bimedial rectus recession with Tutopatch. The left column shows the FBSV before and the right column at the last FU after bimedial rectus recession with Tutopatch. Rows depict individual patients.

A successful outcome of strabismus surgery is frequently defined as an angle under 5∆–10∆ and absence of diplopia in primary position. Following these criteria, all our patients who underwent bimedial tendon elongation with Tutopatch had a successful outcome, as all of them were orthotropic at near and at distance, without diplopia in primary position of gaze and with a reasonable field of binocular single vision.  Figure 4 displays the fields of binocular single vision (FBSV) obtained from the patients with Tutopatch before and after surgery: all patients had double vision in all directions of gaze before surgery, and all had at least a central field of binocular single vision after surgery. This was seen at the 3-month FU in 3 patients and in the other two patients after 11 months (P1) or 18 months (P4). Thus, in tendon elongation with Tutopatch, improvement may be seen over a longer period of time than generally expected.

Table 5 compares our DE to those of the literature. Generally, the DE for recession of the medial rectus is lower than for the inferior rectus muscle. It is even lower following orbital decompression [1]. Comparing our DE to the literature is difficult, as it is often not specified if the DE relates to angles at distance or at near fixation.

In conventional bimedial recession, our DE compared well to the literature: we found a mean DE of 2.95∆/mm at distance and 2.18∆/mm at near fixation. This corresponds to ≈1.69°/mm and 1.25°/mm, respectively. In bimedial recession, the DE has previously been reported as 1.56–1.59°/mm without prior decompression and 1.1–1.3°/mm with prior decompression [1].

The DE in our Tutopatch group was higher than previously reported [10] and also higher than in our patients with conventional bimedial recession (Table 5). This is in contrast to reports of a lower DE in bimedial recessions with Tutopatch compared to conventional bimedial recession [10].

**Figure 5:**
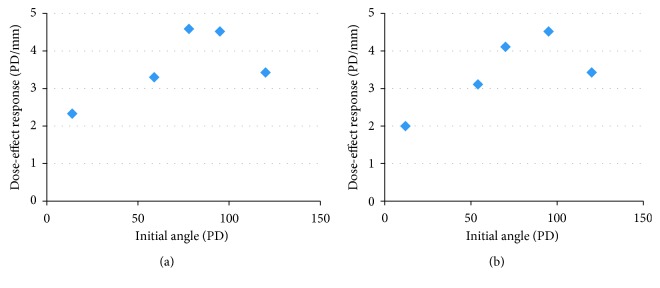
Dose effect at the last follow-up in relation to angle at baseline in the patients with bimedial recession with Tutopatch.

Figure 5 depicts the dependency of the DE on the angle at baseline in our Tutopatch group. The different DE found in our Tutopatch group may be explained by the larger angles at baseline in our Tutopatch group compared to our patients who received conventional bimedial recession. In our Tutopatch group, angles at baseline were also larger than in the patients reported by Eckstein et al. [10], where the maximum angle was 69∆. This is supported by findings in unilateral medial rectus recessions where the DE appears independent of the presurgical angle as long as the latter is <15° [11, 12].

Bimedial rectus recession in TAO resulted in an increase of abduction while decreasing adduction. This held true for bimedial recession with or without tendon elongation. The motility at baseline and consequently at follow-up was better in the group with conventional bimedial recession, reflecting a lesser degree of restrictive disease and also a lesser angle at baseline. Interestingly, both groups showed an initial decrease in adduction at 3 months followed by a slight and similar improvement of adduction and abduction by 0.5–0.8 mm at final follow-up. Overall, good motility was preserved, even in the patients with an extremely large initial angle.

An alternative technique to treat larger squint angles is the use of hang-back sutures. These require the use of nonabsorbable sutures which may be rejected [13]. Time has shown that tendon elongation with Tutopatch has the advantage of allowing the use of absorbable sutures (Table 1) [14]. Also, the Tutopatch graft has been shown to resemble normal tendon on revision surgery [10, 14]. This suggests that in patients requiring further elongation, a revision would appear easier than with hang-back sutures.

A study evaluating the reattachment site of the superior rectus muscle after hang-back recession in rabbits showed that, the higher the amount of recession, the bigger the risk and amount of muscle advancement [15]. In the procedure using Tutopatch, it is not to be expected that the muscle will reattach to the globe posteriorly, as the end of the muscle comes to lie over the interponate (Figure 1). OD-MRI could confirm that the recessed medial rectus was still attached to Tutopatch and not to the globe. In the one patient where scarring was seen, this did not affect the globe. In addition, OD-MRI allowed the appreciation of the elasticity of Tutopatch, allowing for a good passive movement with minimal leash effects.

Strabismus surgery with tendon elongation using Tutopatch is a good alternative to treat large squint angles while preserving good motility. It is worthwhile to expand on this study to include more patients in order to establish more meaningful DE relations. In individual patients, improvement may occur up to 11 months following surgery.

## Figures and Tables

**Table 1:** Patients' characteristics.

Case	Age @ surgery	Radioiodine therapy	Current medication	Orbital decompression	Medial rectus recession with Tutopatch	Sutures
P1	60	No	No	No	OU^b^ Σ 17 mm: OU^b^ MRR^f^ 4 mm + Tutopatch 4.5 mm	Mersilene 5-0

P2A	53	Yes	Eltroxin	1 year prior to first surgery	OU^b^ Σ 6 mm: OU^b^ Tutopatch tendon elongation 3.75 mm OD^c^ anteroposition 1.5 mm	Safil 6-0

P3	43	No	Carbimazol	9 mo prior to surgery	OU^b^ Σ 35 mm: OU^b^ MRR^f^ 4 mm + Tutopatch 13.5 mm	Mersilene 5-0

P4	42	Yes	Levothyroxin-natrium	1.5 years prior to surgery	OU^b^ Σ 18 mm: OD^c^ MRR^f^ 4 mm + Tutopatch 5.5 mm SRR^h^ 3 mm OS^d^ MRR^f^ 3 mm + Tutopatch 5.5 mm	Polysorb 6-0

P5	66	No	Carbimazol	5 mo prior to surgery	OU^b^ Σ 21 mm: OD^c^ Tutopatch tendon elongation 10 mm, IRR^i^ 11 mm OS^d^ Tutopatch tendon elongation 11 mm IRR^i^ 8 mm	Polysorb 6-0

Case	Age @ surgery	Radioiodine therapy	Current medication	Orbital decompression	Conventional bimedial rectus recession	Sutures

P2B^a^	52	Yes	Eltroxin	1 year prior to surgery	OU^b^ Σ 10.5 mm OD^c^ 6 mm OS^d^ 4.5 mm	Not specified

P6	56	Yes	None	15 mo prior to surgery	OU^b^ Σ 7.5 mm	Safil 6-0

P7	35	No	Eltroxin	12 mo prior to surgery	OU^b^ Σ 7 mm OS^d^ SOR^e^	Safil 6-0

P8	50	Yes	Eltroxin	12 mo prior to surgery	OU^b^ Σ 7 mm OD^c^ MRR^f^ 3 mm IOR^g^ OS^d^ MRR^f^ 4 mm IOR^g^	Safil 6-0

^a^Patient 2B is the same patient as 2A who had subsequent bimedial recession with Tutopatch due to a residual angle of 14 PD; ^b^OU = both eyes; ^c^OD = right eye; ^d^OS = left eye; ^e^SOR = superior oblique recession to the nasal side of the superior rectus 4 mm distal to its insertion; ^f^MRR = medial rectus recession; ^g^IOR = inferior oblique recession; ^h^SRR = superior rectus recession; ^i^IRR = inferior rectus recession.

**Table 2:** Recession with Tutopatch: development of horizontal squint angles.

Patient			1	2A (Figure 2)	3 (Figure 3)	4	5
Duration of follow up (months)			84	130	41	36	13

Total recession ∑ OD^b^ + OS^c^ (mm)			17	6	35	18	21

Angle @ distance	Baseline		78^e^	14^e^	120^e^	59^e^	95^e^
	FU^d^	1 week	20^e^	−9^f^	—	25^e^	2.5^f^
		3 months	13^e^	−5^f^	2^f^	12^e^	−12^f^
		Last visit	0	−2^f^	0	4^f^	−11^f^
	DE^g^ (PD/mm)		4.59	2.33	3.428	3.3	4.52

Angle @ near	Baseline		70^e^	12^e^	120^e^	54^e^	95^e^
	FU^d^	1 week	16^e^	−14^f^	−12^e^	20^e^	—
		3 months	3^e^	−16^f^	−14^f^	6^f^	−12
		Last visit	−3^f,h^	−9^f^	−8^f^	−8^f^	−28^f^
	DE^g^ (PD/mm)		4.12	2	3.428	3.11	4.52

^b^OD = right eye; ^c^OS = left eye; ^d^FU = follow-up; ^e^manifest angle; ^f^latent angle; ^g^DE = dose effect; ^h^following correcting the vertical angle.

**Table 3:** Development of motility (mm).

	Tutopatch: P1–P5				Conventional recession: P2B, P6–P8			
	Abduction		Adduction		Abduction		Adduction	
	Mean	Range	Mean	Range	Mean	Range	Mean	Range
Baseline	3.05	0–6^a^	7.95	5–11^b^	6.0	3–8.5	9.375	3.5–11
FU^c^ 3 months	5.25	2–8	5.3	2.5–7.5	6.83	5–9	8	7–9.5
FU^c^ last visit	5.7	2.5–8.5	5.9	2–9	7.56	4.5–10	8.81	7.5–10

^a^Abduction assumed to be zero in P3 and P5 because midline could not be reached; ^b^adduction measured as the possible motility from the starting point, not primary position; ^c^FU = follow up.

**Table 4:** Conventional bimedial recession: development of horizontal squint angles.

Patient			2B^a^	6	7	8
Duration of follow-up (months)			7.5	47	81	87

Total recession ∑ OD^b^ + OS^c^ (mm)			10.5	7.5	7	7

Angle @ distance	Baseline		35^e^	30^e^	18^e^	25^e^
	FU^d^	1 week	18^e^	4^f^	7^e^	5^e^
		3 months	17^e^	0	14^f^	3^e^
		Last visit	14^e^	2^f^	^2e,h^	0.5^e,h^
	DE^g^ (PD/mm)		2	4	2.29	3.5

Angle @ near	Baseline		33^e^	23^e^	12^e^	16^e^
	FU^d^	1 week	12	0	—	0
		3 months	14^e^	-2^f^	6^f^	3^f^
		Last visit	12^e^	2^f^	2^e,h^	0.5^e,h^
	DE^g^ (PD/mm)		2	3.06	1.43	2.21

^a^Patient 2B is the same patient as 2A who had subsequent bimedial recession with Tutopatch due to a residual angle of 14 PD; ^b^OD = right eye; ^c^OS = left eye; ^d^FU = follow-up; ^e^manifest angle; ^f^latent angle; ^g^DE = dose effect; ^h^underlying microesotropia with latent esophoria.

**Table 5:** Dose effect (PD/mm): comparison with the literature.

Method		Our results	Results in literature^a^
Recession with Tutopatch	Bilateral M. rectus medialis	@ distance: 3.63	1.57–1.75^b,c^
		@ near fixation: 3.43	1.39–1.57^b,d^
	Unilateral M. rectus inferior	—	3.46–3.58^b,c^

Conventional recession	Bilateral M. rectus medialis	@ distance: 2.95	2.72–2.77^c,e^
		@ near fixation: 2.18	1.92–2.27^b,c^
	Unilateral M. rectus inferior	—	3.49^b,c^

^a^Not specified if angle at near fixation or distance, degrees converted to prism diopters (rate: °/0.5729); ^b^with prior decompression; ^c^Eckstein, Schittkowski et al.[1]; ^d^Eckstein et al. [10]; ^e^without prior decompression.

## Supplementary Materials

Supplementary 1 Video 1: patient 1: supplementary video 1 shows the motility of patient 1 as seen on OD-MRI. In adduction, the contracting belly of the medial rectus muscle is seen behind the eye with the thin tendon extension attached well anterior of the equator. There is free movement without scarring and no leash effect on abduction.

Supplementary 2 Video 2: patient 2: OD-MRI of patient 2 shows smooth horizontal motility without apparent restrictions (supplementary video 2).

Supplementary 3 Video 3: patient 4: OD-MRI (supplementary video 3) shows the swollen orbital tissue (OS > OD) displaced medially following medial and lateral wall decompression. In agreement with the Kestenbaum measures, adduction is impaired in OD compared to OS, and abduction is impaired in OS compared to OD.

Supplementary 4 Video 4: patient 5: OD-MRI supplementary videos 4–6 show the dramatically improved horizontal and vertical motility. The swollen orbital tissue (OS > OD) is displaced nasally following medial wall decompression. On the horizontal OD-MRI (supplementary video 4), the asymmetry of the Tutopatch elongation (OS > OD) can be appreciated. In contrast to P1, P2, and P4, a second proximal thickening in the area of Tutopatch is indicative of adhesions (scar tissue) to the surrounding connective tissue in the area of the suture between Tutopatch and the muscle. As a consequence, abduction is noticeably impaired and comes to a more abrupt stop, which may also reflect the greater amount of orbital fat.
